# Non-Destructive Analysis of the Internal Anatomical Structures of Mosquito Specimens Using Optical Coherence Tomography

**DOI:** 10.3390/s17081897

**Published:** 2017-08-17

**Authors:** Naresh Kumar Ravichandran, Ruchire Eranga Wijesinghe, Seung-Yeol Lee, Kwang Shik Choi, Mansik Jeon, Hee-Young Jung, Jeehyun Kim

**Affiliations:** 1 School of Electronics Engineering, College of IT Engineering, Kyungpook National University, 80 Daehak-ro, Buk-gu, Daegu 41566, Korea; nareshr.9169@gmail.com (N.K.R.); eranga@knu.ac.kr (R.E.W.); jeehk@knu.ac.kr (J.K.); 2 School of Applied Biosciences, Kyungpook National University, 80 Daehak-ro, Buk-gu, Daegu 41566, Korea; leesy1985@gmail.com (S.-Y.L.); heeyoung@knu.ac.kr (H.-Y.J.); 3 School of Life Sciences, Kyungpook National University, 80 Daehak-ro, Buk-gu, Daegu 41566, Korea

**Keywords:** mosquito, optical coherence tomography, internal morphological analysis

## Abstract

The study of mosquitoes and analysis of their behavior are of crucial importance in the on-going efforts to control the alarming increase in mosquito-borne diseases. Furthermore, a non-destructive and real-time imaging technique to study the anatomical features of mosquito specimens can greatly aid the study of mosquitoes. In this study, we demonstrate the three-dimensional imaging capabilities of optical coherence tomography (OCT) for structural analysis of *Anopheles sinensis* mosquitoes. The anatomical features of *An. sinensis* head, thorax, and abdominal regions, along with the morphology of internal structures, such as foregut, midgut, and hindgut, were studied using OCT imaging. Two-dimensional and three-dimensional OCT images, used in conjunction with histological images, proved useful for anatomical analysis of mosquito specimens. By presenting this work as an initial study, we demonstrate the applicability of OCT for future mosquito-related entomological research, and also in identifying changes in mosquito anatomical structure.

## 1. Introduction

Mosquitoes are recognized as one of the major vectors of tropical diseases such as malaria, dengue, chikungunya, West Nile virus, yellow fever, lymphatic filariasis, Japanese encephalitis, Zika fever, and many other blood-transmittable diseases [1]. These vector-borne diseases are considered major threats for vast populations worldwide. Approximately 212 million cases of malaria occurred in 2015, resulting in the death of approximately 429,000 people [2]. Many cases of Zika virus infection have also been reported worldwide. On the basis of recent findings, it has been concluded that Zika virus infection in pregnancy can be a cause of microcephaly [3]. Similarly, West Nile fever affects various mammalian species, including humans, and reptilian species [4]. Many of the mosquito-transmitted diseases pose high-level risks if not treated promptly. The pathogens causing these diseases are transmitted from the mosquito to the host during blood feeding. Thus, research on mosquitoes and the associated pathogen transmission plays a major role in controlling the ever-growing transmission of disease via mosquito bites. Many medical and research teams have been studying the feeding habitats [5,6], probing behavior [7], feeding persistence [8], life cycle, growth stages, and other important aspects of mosquitoes [9], thereby contributing to a reduction in the rapidly growing prevalence of mosquito-borne disease.

Study of the organs of mosquitoes, such as the proboscis, head, abdomen, thorax, midgut, and circulatory system provides extensive necessary information for entomological researchers. To date, many techniques have been utilized for the study and analysis of internal structures. Techniques such as micro-computed tomography [10], microscopic and histological analysis [11,12], and X-ray imaging [13] have been implemented for mosquito studies. However, these techniques require time-consuming sectioning processes, a complex system design, or a long imaging acquisition time. Moreover, the cost of these techniques may be prohibitive for frequent implementation. Thus, a rapid bio-imaging technique would be of considerable advantage to researchers for studying the internal organs of mosquitoes.

Optical coherence tomography (OCT) is an interferometric imaging modality that uses a broadband light source to provide high-resolution cross-sectional images in real-time without sample destruction [14], which is difficult to achieve using conventional methods. Applications of OCT have been demonstrated in various fields of research, including ophthalmology [15,16], otorhinolaryngology [17,18], industrial inspection [19], agronomical studies [20,21,22], and entomological studies [23,24,25,26]. Recently, OCT has been applied in various entomological studies, such as those developing neural morphological analysis and cardiac dynamics in *Xenopus laevis* [25,27], age estimation of *Calliphora vicina* pupae [23], and arrhythmia caused by a *Drosophila Tropomyosin* mutation [28]. To date, however, there have been no reports on the use of OCT as a tool for studying the internal organs of mosquito specimens.

In this study, we undertook a morphological analysis of *Anopheles sinensis* mosquitoes using spectral domain optical coherence tomography (SD-OCT). Thus, we have demonstrated the potential application of OCT for the identification of internal structure morphology in mosquito specimens. This was a feasibility study and by analyzing the results of the two-dimensional (2D) and three-dimensional (3D) OCT images using histological images, we have demonstrated the advantages of using OCT for analysis of mosquito organs and tissue structures for future studies.

## 2. Materials and Methods

### 2.1. Mosquito Species Identification

Mosquito samples were collected in 2014 at Majeong-ri, Paju-si (37°52′53.52″ N, 126°45′24.89″ E), Korea, using a black light trap. Specimens were identified using the molecular method described by [29], after DNA extraction from a single leg using a genomic DNA extraction kit (Bioneer Inc., Daejeon, Korea). The polymerase chain reaction (PCR) conditions were as follows: The 12.5-μL PCR reaction mixture contained 0.5 μL genomic DNA of an individual mosquito, 0.5 units of Taq polymerase, 0.4 μM of each primer (see [29] for the primer information), 1.5 mM MgCl_2_, 0.2 mM of each dNTP, and 1X PCR buffer. The PCR cycling conditions were as follows: initial denaturation at 94 °C for 3 min, followed by 30 cycles at 94 °C for 30 s, 55 °C for 30 s, 72 °C for 2 min, and a final extension at 72 °C for 7 min. The PCR products were electrophoresed using ethidium bromide added in 2.5% agarose gel and visualized under a UV illuminator.

### 2.2. Mosquito Specimen Preparation for OCT Imaging

**Figure 1:**
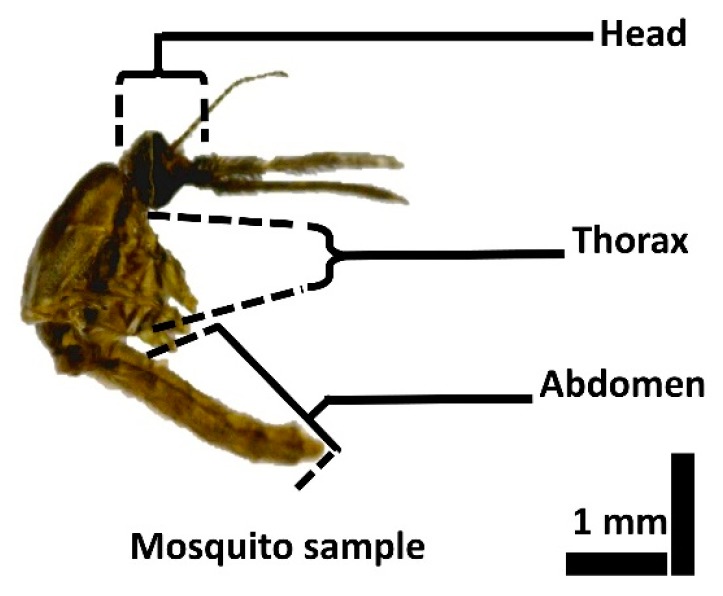
Microscopic image of an adult mosquito specimen.

A representative image of a mosquito specimen is shown in Figure 1. To anesthetize the specimens, cotton dipped in ethyl acetate was suspended in a container in which the specimens were kept. When completely anesthetized, the mosquitoes were carefully separated and placed in a petri dish. Prior to placement, the petri dishes were washed with sterile distilled water and then dried. The experimental environment was maintained at 23 °C and 50% humidity. OCT imaging of the specimens was carried out immediately after the specimens were anesthetized. For experimental convenience, the wings and the legs of the specimens were clipped carefully without damaging other parts of the specimen. All the specimens used for the experiment were fully grown adults, and their average size was ~4 mm in length.

### 2.3. Specimen Preparation for Histological Analysis

The selected specimens were used for histological analysis to enable a comparative analysis with the obtained 2D OCT images. The specimens were fixed in 2% paraformaldehyde and 2.5% glutaraldehyde in 0.05 M sodium cacodylate buffer for 24 h under vacuum. Thereafter, the specimens were washed in sterile distilled water and dehydrated in a graded series of absolute ethanol (30%, 50%, 70%, 80%, 90% and absolute ethanol) for 30 min. These dehydrated specimens were infiltrated with propylene oxide and then embedded in Spurr’s resin. Afterwards, the specimen was polymerized in Spurr’s resin for 24 h at 70 °C. Finally, the samples were accurately sectioned using an ultra-microtome (MT-7000; RMC, Tucson, AZ, USA), 2 μm in thickness. The individual sections were stained with 2% methylene blue and observed under a light microscope (BX-50, Olympus, Japan) to analyze the histological image.

### 2.4. SD-OCT System Setup

**Figure 2:**
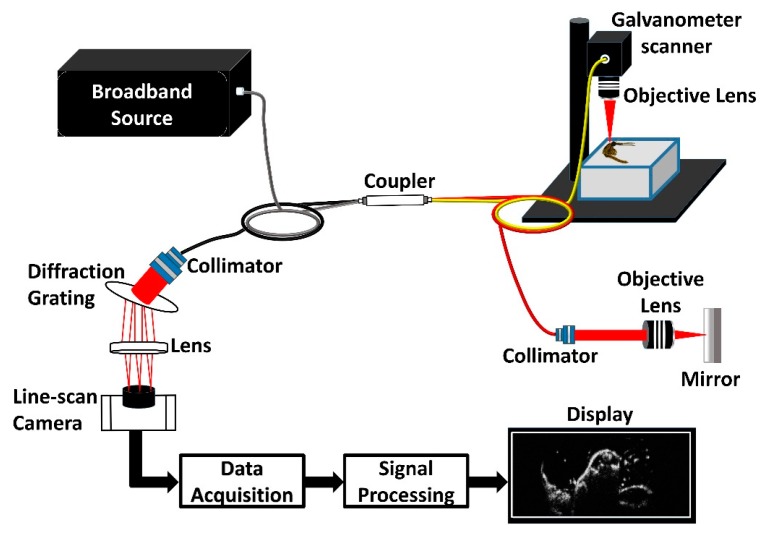
Representation of the spectral domain optical coherence tomography (SD-OCT) setup used in the present study.

Figure 2 is a schematic representation of the high-resolution spectral domain optical coherence tomography (SD-OCT) system that was utilized in the present study. The laboratory-built SD-OCT system comprises a broad bandwidth laser source (BroadLighters T-850-HP; Superlum Diodes Ltd., Carrigtwohill, Co., Cork, Ireland) with a central wavelength of 860 nm and a full width at half maximum of 165 nm. The optical power of the laser source is 12 mW. The output from the laser source is connected to a wideband fiber optic coupler of ratio 75:25 (TW850R3A2, Thorlabs, Inc., Newton, NJ, USA). Of the optical power from the coupler, 25% is directed to the reference arm and the remaining 75% is directed to the sample arm.

The reference arm consists of a collimator (F280APC-850, Thorlabs, Inc., Newton, NJ, USA), a plan apochromatic objective lens of 10X magnification (10X M Plan APO, Edmund Optics, Barrington, NJ, USA), and a high reflective mirror (ME1-G01, Thorlabs, Inc., Newton, NJ, USA). The sample arm beam is collimated using a collimator (F280APC-850, Thorlabs, Inc., Newton, NJ, USA) and the collimated beam is scanned on the specimen using a galvanometer scanner (GVS002; Thorlabs, Inc., Newton, NJ, USA) and focused using a plan apochromatic objective lens of 10X magnification (10X M Plan APO, Edmund Optics, Barrington, NJ, USA). The objective lens is utilized to improve the lateral resolution of the optical system for insect imaging. The backscattered and interfered signals are detected using a spectrometer setup, consisting of a collimator (F810APC-842, Thorlabs, Inc., Newton, NJ, USA) a transmission-type diffraction grating (HD1800 lpmm, Wasatch Photonics, Durham, NC, USA), an achromatic lens (AC508-100-B; Thorlabs, Inc., Newton, NJ, USA), and a 4096-pixel line scan camera (spL4096-140 km; Basler AG, Ahrensburg, Germany). The theoretical maximum achievable lateral resolution with this configuration is 5.38 µm, for a sample arm beam diameter of a maximum 3.9 mm and objective lens’s focal length of 20 mm. This beam diameter value is the ideal value, which is difficult to attain. Due to alignment and optical limitations, the attained lateral resolution of the built system is 10 µm, which was sufficient for the visualization of mosquito internal organs in the lateral direction. Thus, the obtained axial and lateral resolution of the built system is 4 µm and 10 µm, respectively. To obtain a real-time 2D OCT image, 500 adjacent A-scans (axial depth scan) of the specimen surface are scanned laterally and combined. Similarly, to obtain a 3D volumetric OCT image, 500 adjacent 2D images are combined. The depth dimension in a 2D image was 2 mm and the scan range was 4 mm × 4 mm. The system was capable of producing 32 frames per second, thus enabling a 2D image acquisition at 31.25 milliseconds and one volumetric image acquisition takes around 15 s. The signal to noise ratio of the built system is 106 dB. The calculated total Rayleigh range was 365 µm, which was sufficient for the mosquito specimen organ analysis. Also, the depth roll-off was less than 5 dB which was calculated by point spread function, thus making the system capable of volumetric mosquito imaging. However, for convenience in analyzing the structures at different depths, the focus position was changed as per the region of interest.

## 3. Results and Discussion

### 3.1. Volumetric 3D OCT Image Analysis of Mosquito Specimens

2D cross-sectional OCT images were acquired from the head, thorax, and abdominal regions of a mosquito body. Cross sections of the mosquito internal organs, showing the internal tissue structures, were studied by obtaining adjacent 2D-OCT images of all three regions. Using these prominent structures in 2D cross-sectional images, the 3D OCT images were obtained by focusing on these internal structures. The 3D OCT images were obtained by taking adjacent 2D-OCT images and combining these images using volume-rendering software.

**Figure 3:**
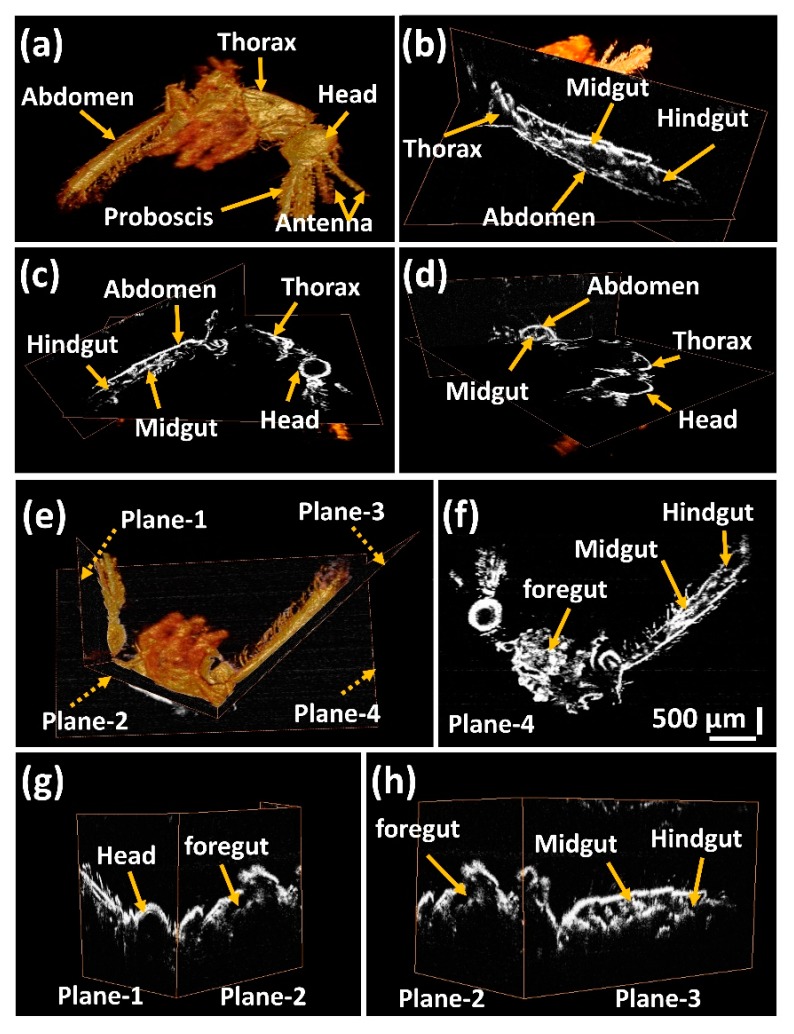
Three-dimensional optical coherence tomography (3D OCT) image of a mosquito specimen, and the orthogonal image section planes 1, 2, 3, and 4 showing the continuity of the internal structures in the mosquito specimen. (**a**–**d**) are orthogonal-sectioned images of specimen at different depths. (**e**–**h**) are orthogonal-sectioning planes 1, 2, 3, and 4 showing the continuity of internal structures. Refer to the supplementary media file for internal structure analysis at different depths.

A volumetric 3D OCT image of a mosquito specimen is shown in Figure 3a with the proboscis, antenna, head, thorax, and the abdomen indicated. Figure 3b–d show the orthogonal-sectioned images of the 3D images, revealing the internal morphology of the head, thorax, and abdominal regions of the mosquito specimen. The midgut and hindgut are shown in Figure 3c,d. In addition, individual sections of the head, thorax, and abdominal regions are shown in Figure 3e using orthogonal sectioning planes in the 3D volumetric image, which are represented by dotted arrows with representations of plane-1, plane-2, plane-3, and plane-4. The individual sectioning is shown in Figure 3f–h. The continuity of the internal organs can be seen using these sectioning planes. 

Figure 3f is an orthogonally sectioned (using image section plane-4) image of the 3D OCT image in the sagittal plane section at the mid-part of the mosquito body. Figure 3g shows two sagittal planes sectioned in the head region (using image section plane-1) and the thorax region (using image section plane-2), as indicated in Figure 3e. Similarly, Figure 3h shows the two sagittal planes sectioned in the thorax region (using image section plane-2) and the abdominal region (using image section plane-3), as indicated in Figure 3e. The foregut, midgut, and hindgut are clearly identifiable through the individually sectioned images as shown below. The orthogonal image section planes 1–3 help to visualize the continuity of the internal structures in the specimen, showing the entire cross-section for easier analysis of the mosquito specimen.

### 3.2. Histological Image Comparison Using OCT Images of Mosquito Specimens

**Figure 4:**
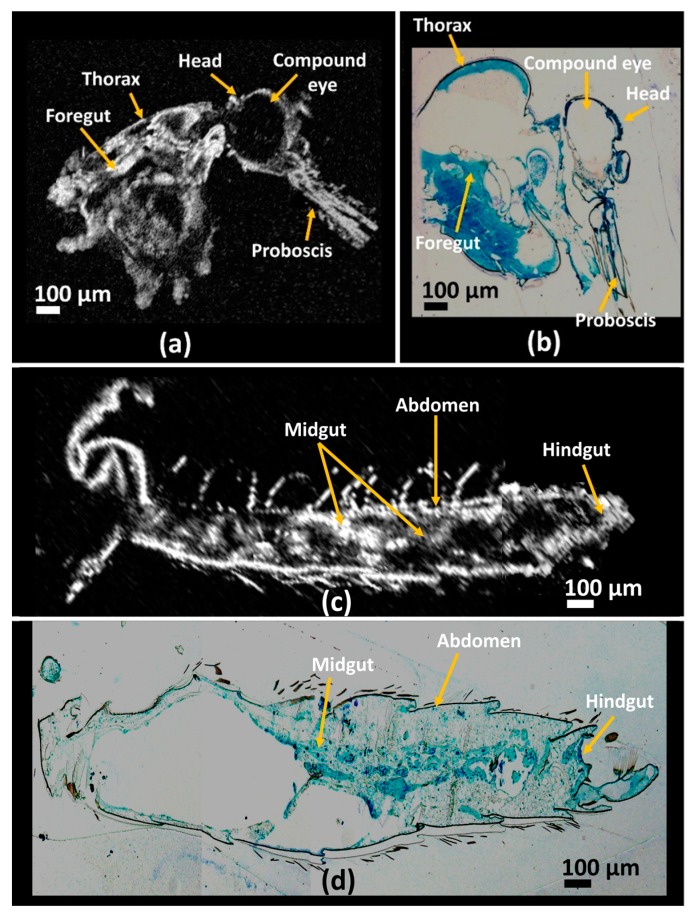
Comparative analysis of optical coherence tomography (OCT) images with histological images of a mosquito specimen. (**a**,**c**) are orthogonal-sectioned OCT volumetric image at head, and thorax regions and abdomen region respectively. Images (**b**,**d**) are the corresponding histology sectioned microscopic image of the specimen at head, and thorax region and abdomen region.

Best-fit mosquito specimens underwent histological sectioning, as previously mentioned in Section 3.2. The histological sectioning of specimens was performed in the sagittal direction for better visualization of the internal structures of the specimens. For comparison of OCT images with the obtained histological images, 3D volumetric images were orthogonally sliced with post-image analysis software. After orthoslicing of the 3D volumetric OCT image, the analysis was performed in the same direction as histological sectioning. By visualizing the orthosliced images at different depths, the most correlative OCT image was selected for image comparison with the histological image. Figure 4a shows an orthosliced OCT image of the mosquito specimen, depicting the head and thorax regions of the specimen, and Figure 4b is the histological image of the head and thorax regions of a mosquito specimen. Foregut, head, proboscis, compound eye, and other internal structures in the thorax region are seen in the OCT image, which are consistent with the histological image. Figure 4c is the OCT image of a mosquito abdomen region and Figure 4d is the respective histological image of the mosquito specimen in the abdominal region. The midgut and hindgut along with other internal abdominal structures are visible in the OCT image, which can be correlated with the obtained histological image. For better image analysis, the focusing point was varied depending on the region of interest. For instance, Figure 4a,c were taken from two volume rendered images orthosliced where Figure 4a is focused on the thorax region and Figure 4c is focused on the abdomen region of the same sample. This was necessary as the sample was curved and its shape was not uniform.

## 4. Conclusions

Through our study, we have demonstrated the benefits of high-resolution implementation and the three-dimensional imaging capability of OCT for structural analysis of *An. sinensis* mosquitoes. By using a high-resolution customized OCT system, internal structures, such as foregut, midgut, and hindgut, were clearly visualized using OCT imaging, supported by histological image verification. Due to the importance of vector-borne diseases, studies on mosquitoes are considered essential; however, to date, initial morphological studies and analysis of the anatomical structures of mosquitoes using OCT have not been performed. The results presented in this study will be helpful for researchers in non-destructive visualization of internal anatomical structures of *An. sinensis* mosquitoes and structural features and continuity of the foregut in the thorax region and midgut in the abdomen region will be helpful for researchers in respective fields of interest. We utilized an 860 nm centered wavelength source to obtain a better resolved image which was suitable for visualizing the internal organs in the respective region of interest in the specimens. Also, in future applications, OCT imaging incorporated with structural organ tracing can be useful for individual visualization of anatomical structures in real-time. Furthermore, by utilizing a 1300 nm range optical coherence microscopic system, deeper penetration can be achieved with resolutions on a par with histology images. Further, owing to the high-resolution and real-time in vivo imaging capability of OCT, future in vivo studies on different mosquito vectors of disease-causing pathogens may help researchers to better understand how diseases are transmitted via mosquito bites.

## Supplementary Materials

The following are available online at http://www.mdpi.com/1424-8220/17/8/1897/s1, Video S1: Internal structure analysis of SD-OCT 3D image of a mosquito specimen at different depths.
